# Toll-like receptors in inflammatory bowel diseases: A decade later

**DOI:** 10.1002/ibd.21282

**Published:** 2010-04-12

**Authors:** Elke Cario

**Affiliations:** Division of Gastroenterology & Hepatology, University Hospital of Essen, and Medical School, University of Duisburg-EssenEssen, Germany

**Keywords:** Toll-like receptor, inflammatory bowel disease, review, innate immunity, host defense, intestinal mucosa, Crohn's disease, ulcerative colitis

## Abstract

Differential alteration of Toll-like receptor (TLR) expression in inflammatory bowel disease (IBD) was first described 10 years ago. Since then, studies from many groups have led to the current concept that TLRs represent key mediators of innate host defense in the intestine, involved in maintaining mucosal as well as commensal homeostasis. Recent findings in diverse murine models of colitis have helped to reveal the mechanistic importance of TLR dysfunction in IBD pathogenesis. It has become evident that environment, genetics, and host immunity form a multidimensional and highly interactive regulatory triad that controls TLR function in the intestinal mucosa. Imbalanced relationships within this triad may promote aberrant TLR signaling, critically contributing to acute and chronic intestinal inflammatory processes in IBD colitis and associated cancer. (Inflamm Bowel Dis 2010)

Perturbed homeostasis between commensal bacteria and mucosal immunity serves as a critical determinant in the development of gut inflammation in inflammatory bowel disease (IBD) in the genetically susceptible individual.^1^,^2^ Innate immune cells must exert a rigorous process of rapid and precise discrimination between “self” and “nonself” based on the recognition of broadly conserved molecular patterns by so-called pattern recognition receptors (PRRs).^3^ Toll-like receptors (TLRs), a class of transmembrane PRRs, play a key role in induction of pro/antiinflammatory genes and control of adaptive immune responses.^4^,^5^ In 2000, differential modulation of TLRs in the intestinal mucosa was first described in IBD.^6^ Since then, much progress has been made in defining the cell-specific effects and mechanisms through which TLRs mediate recognition and sorting of the broad spectrum of diverse products of the luminal microbiota and how aberrant TLR modulation may contribute to the development of IBD. This review focuses on recent advances in our understanding of the complex involvement of regulatory effects (environmental factors, gene variants, and mucosal immunity) on intestinal TLR function in IBD pathogenesis (Fig. 1). Within a healthy host, TLR signaling drives basal immune mechanisms essential for protecting host barrier integrity and maintaining commensal composition and tolerance. However, within a susceptible individual, aberrant or dysfunctional TLR signaling may impair commensal-mucosal homeostasis, thus contributing to amplification and perpetuation of tissue injury and consequently leading to chronic inflammation in IBD.

**FIGURE 1:**
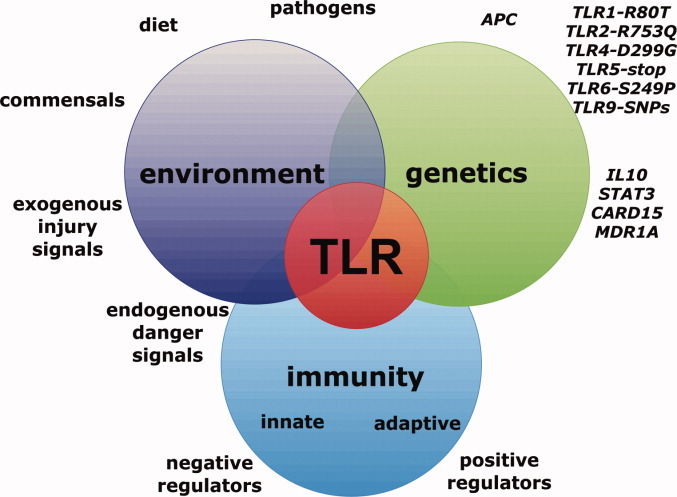
Environment, genetics, and host immunity form a multidimensional and highly interactive regulatory triad that controls TLR function in the intestinal mucosa. Multiple factors may positively or negatively regulate TLR signaling.

## STRUCTURE AND SIGNALING

TLRs comprise a class of 13 mammalian type I transmembrane glycoproteins (10 in humans and 12 in mice) which all contain multiple leucine-rich repeat motifs (LRR) in the large, divergent ectodomain and a highly conserved region in the short intracellular tail, called the Toll-interleukin-1 receptor (TIR) domain. The TIR domain consists of sites essential for interaction between homo- or heterodimeric TLR subunits as well as recruitment of cytoplasmic adapter proteins to initiate downstream signaling cascades. The TIR domain is not unique to TLRs and can also be found in receptors of the IL-1, IL-18, and IL-33 families, implying evolutionary convergence into common immune responses to distinct inflammatory stimuli.

TLRs recognize alarm signals that can be classified into microbiota-/viral-associated (commensal/pathogen) and damage-associated (endogenous/exogenous) molecular patterns. Molecular signatures of different classes of microorganisms or features include, e.g., lipopeptides: TLR2; viral-derived dsRNA: TLR3; lipopolysaccharide: TLR4; flagellin: TLR5 and CpG DNA: TLR9. Ligand binding elicits receptor activation through conformational changes. To date, at least five different adaptor proteins have been identified: MyD88, Mal/TIRAP, TRIF/TICAM-1, TRAM/Tirp/TICAM-2, and SARM. All TLRs, except TLR3, may signal through the adaptor protein MyD88, while TLR4 uses both MyD88-dependent and MyD88-independent pathways. Engagement of MyD88 activates a series of signaling modules, including IRAK, TRAF6, and TAK1, ultimately leading to activation of transcription factors (NF-κB, AP-1, Elk-1, CREB, STATs, or IRF) (reviewed, e.g., in Refs.^7^,^8^). Besides MyD88, TRAF6 functions downstream as another common signaling checkpoint of several pathways and thus interconnects the IL-1R/TLR and TNFR superfamilies. Subsequent transcriptional activation of unique and common TLR target genes encoding pro- and antiinflammatory cytokines and chemokines as well as the induction of costimulatory molecules control the activation of antigen-specific and nonspecific adaptive immune responses by lamina propria cells. All of these various downstream effects are critically involved in protection of host homeostasis through control of milieu influences.

## EXPRESSION PATTERN IN HEALTH AND IBD

TLRs are inducibly or constitutively expressed in different combinations throughout the whole gastrointestinal tract by a wide variety of cell types, including the four principal intestinal epithelial cell (IEC) lineages (absorptive enterocytes,^9^–^14^ Paneth cells,^15^,^16^ goblet cells,^17^ enteroendocrine cells^18^,^19^), subepithelial myofibroblasts,^20^,^21^ and various professional immune cell subsets within the intestinal lamina propria (such as monocytes/macrophages,^22^,^23^ dendritic cells [DCs],^24^–^26^ and CD4+ T cells^27^,^28^). Specific cell types express individual patterns of TLRs at different anatomical sites. For instance, when differentiating into immature DCs, monocytes progressively lose the expression of some TLRs, but gain the expression of others.^29^ Bone-marrow-derived CD11c+ DC express substantial levels of TLR4 to rapidly recognize detrimental pathogenic threats.^30^ In contrast, lamina propria CD11c+ DCs do not express TLR4 to maintain hyporesponsiveness to omnipresent lipopolysaccharide (LPS) in the gut lumen.^25^,^26^ Distinct TLR expression patterns may thus reflect different functional necessities of TLR ligand recognition at different strategic locations.

Based on the optimal sites of ligand recognition and binding, TLRs are strategically localized either on the cell surface (TLR1/2/4/5/6) or in intracellular compartments (TLR3/7/8/9). Intestinal epithelial TLR localization and ligand responsiveness may be critically modified by state of cellular activation,^9^,^31^ polarity,^32^ and differentiation.^33^ While TLR2 and TLR4 are preferentially localized at the apical pole of differentiated enterocytes in vitro,^33^ TLR5 is stably expressed at the basolateral pole of the intestinal epithelium in vitro,^11^ the major site of action for *Salmonella*-translocated flagellin upon injury. Although the presence of MD-2 retains TLR4 to the cellular surface localization,^34^ TLR4 is capable to shuttle its cargo LPS between plasma membrane and endosomal structures,^33^ which are part of the Golgi apparatus.^35^,^36^ In addition, distinct TLRs may interact with the endoplasmatic reticulum (ER) through accessory molecules, such as TLR3/7/9 via Unc93B,^37^ TLR1/4 via PRAT4A,^38^ or TLR4/9 via heat shock protein 96.^39^

In the normal intestine, TLR2 and TLR4 are present only in small amounts on IEC and lamina propria mononuclear cells (LPMNCs) in vivo, minimizing recognition of the environment and maintaining a basal state of activation.^6^,^10^,^12^–^14^,^23^ TLR inhibition acts to avoid inappropriate activation despite the omnipresent microbiota. Cellular mechanisms, like compartmentalization or differential activation, as well as several negative regulators, have been found to attenuate or abrogate TLR activation in the intestinal mucosa. Once host threats are encountered, these inhibitory mechanisms can be switched off, and positive regulators allow TLR signaling to elicit important immune responses, in the attempt to eliminate the danger. But sustained TLR hyperactivation may provoke chronic inflammation in IBD. TLR4 is significantly increased in primary IEC and LPMNC throughout the lower gastrointestinal tract in active disease of both human Crohn's disease (CD) and ulcerative colitis (UC),^6^ maximizing responsiveness to the environment and reflecting an aberrant state of activation.

TLR4 signaling requires three accessory molecules, CD14, LBP, and MD-2. Under healthy conditions, expression of this receptor complex is generally low in the intestinal mucosa,^22^,^31^,^40^,^41^ but significantly upregulated in various cell subsets in either nonactive and/or active human IBD colitis.^40^–^42^ MD-2/TLR4 upregulation could also result from ligands other than abundant LPS. T-cell-derived cytokines, such as interferon gamma (IFNγ) and tumor necrosis factor alpha (TNF-α), which play significant pathophysiological roles in triggering IBD, have been found to upregulate intestinal epithelial TLR4 expression in vitro.^31^,^43^ Changes in the commensal composition in the genetically susceptible host may result in aberrant TLR4 hyperresponsiveness of the intestinal mucosa.^44^ But receptor upregulation may also reflect functional loss of immune responses.

## ENVIRONMENT

### Commensals

Commensal–host interactions are based on symbiotic mutualism in which both partners benefit. The constant exposure of the intestinal mucosal surface to commensal-derived TLR ligands induces a basal state of activation of downstream signaling pathways that ensures mucosal homeostasis through limited inflammatory responses and accelerated restitution and healing in the healthy intestine. Commensal composition and tolerance represent essential mechanisms of maintaining hyporesponsiveness of the intestinal immune system.

The composition of the commensal microbiota depends on host immunity, genetics, and environment. Host immunity contains the commensal composition to avoid excessive antigen signals. Emerging evidence reveals that host-derived antimicrobial peptides, predominantly Paneth cell α-defensins, have a key role in determining the commensal composition.^45^ In this function, innate immunity is essentially complemented by adaptive immune mechanisms. Bacterial overgrowth and mucosal penetration are minimized by IgA production, which is induced either by commensal-loaded DCs^46^ or IEC.^47^ (CD4+CD25+Foxp3+) T regulatory (Tregs) critically coordinate cellular IgA responses in the intestinal mucosa.^48^ TLR stimulation of IEC may induce active DC sampling^49^ and production of APRIL in neighboring DCs, which then stimulate naive IgD+ B cells to express mucosa-protective IgA_2_ in the presence of IL-10.^47^ TLR ligation of IEC may also promote IgG and IgA class switching via BAFF, which is self-controlled through SLPI.^50^ In TLR deficiency, adaptive immunity (and/or other PRRs) can step into the breach and restore effective bacterial clearance by high production of commensal-specific IgG antibodies.^51^

In return, the composition of the commensal microbiota actively shapes mucosal and systemic immune homeostasis of the host at multidimensional levels. The presence of commensals modulates TLR expression in the intestinal mucosa.^52^ The complexity of the commensal composition is critical in augmenting protective mucosal immunity.^53^ Certain commensal species help to maintain an immunoregulatory environment through antiinflammatory effects and inhibition of specific intracellular signal transduction pathways in the intestinal mucosa.^54^,^55^ Several studies have recently demonstrated the importance of the commensal composition in orchestrating the TH17↔Treg balance within the lamina propria.^56^–^58^ Any disturbance in this fine-tuned partnership between commensals and the host cells may impair their mutually beneficial interactions. Distinct perturbations and alterations in the commensal composition may deregulate mucosal immune responses. However, in the genetically immunoincompetent host, the commensal composition may shift and turn pathogenic. Aberrant expansion of selected commensals^59^ may launch tissue-destructive host responses and drive colitis.^60^

Changes in the commensal composition may differentially modulate mucosal TLR responsiveness, thus subverting immune responses to a predominantly proinflammatory phenotype. IBD patients contain abnormal compositions of the intestinal microbiota, characterized by reduced bacterial diversity,^61^ temporal instability,^62^ and depletion of distinct commensal species (members of the phyla Firmicutes and Bacteroidetes).^63^ The latter includes a lower proportion of *Faecalibacterium prausnitzii*, an antiinflammatory commensal that counterbalances dysbiosis.^64^ CD patients are predisposed to become colonized with facultative-pathogenic commensals, such as the adherent invasive *E. coli* (AIEC) that harbors various virulence factors involved in adhesion and invasion of the IEC barrier.^65^ Chitinase 3-like-1 may play an important pathogenic role in mediating enhancement of commensal adhesion to IEC in IBD.^66^ Oral infection of mice with flagellated AIEC^67^ induces a significant increase of TLR5 expression in the gut that is associated with colitis aggravation.^68^ Host defense mechanisms, especially antimicrobial activities through defensins that limit bacterial invasion and expansion, are severely impaired in IBD. Decreased expression of Paneth cell α-defensins has recently been associated with increased susceptibility to develop CD ileitis.^69^ MyD88-dependent signaling, presumably via TLR2/4,^19^,^70^,^71^ is crucial for limiting mucosal adherence and penetration of commensals through production of Paneth cell α-defensins^16^ and RegIIIγ.^72^,^73^ Several causal scenarios are plausible in IBD pathogenesis, but remain to be directly proven: Genetic defects and/or aberrant immune-mediated modulation of specific TLRs may diminish antimicrobial activities and disturb bacterial clearance, leading to a colitogenic commensal composition. Changes in the commensal composition may subvert the mucosal innate immune system, leading to TLR-mediated hyper- or hyporeactive immune responses. Dysbiosis may allow facultative-pathogens to submerse, avoiding effective TLR recognition and bactericidal activation. Taken together, it will be important to define the mechanisms in detail: 1) how TLR signaling shapes the antimicrobial tone of the intestinal immune system, in this way critically influencing the commensal composition, and 2) how IBD-related changes in the commensal composition and facultative-pathogenic commensals may functionally skew TLR signaling in the genetically susceptible host.

Several negative control mechanisms that ensure tolerance to abundant resident microbiota and regulated activation via TLRs in the intestinal mucosa have recently been described (detailed review in Ref.^74^): decreased surface receptor expression which limits frontline recognition,^6^,^10^,^41^,^43^ high expression levels of the downstream signaling suppressor Tollip, which inhibits IRAK activation,^13^ ligand-induced activation of PPARγ (peroxisome proliferator-activated receptor γ), which uncouples NF-κB-dependent target genes in a negative feedback loop,^55^,^75^ negative regulation of proinflammatory IL-1R/TLR4 signaling through SIGIRR (single immunoglobulin IL-1R-related molecule; also known as TIR8), which abolishes exaggerated immune responses to commensal bacteria in colitis,^76^–^78^ ubiquitylation of key TLR signaling components via ubiquitin-editing enzymes, such as A20,^79^–^81^ or E3 ubiquitin-protein ligases, such as TRIAD3A,^82^ and selective induction of transcriptional repressors, such as Bcl-3, which limits proinflammatory responses via NF-κB.^83^ Digestive enzymes, such as intestinal alkaline phosphatase^84^ or trypsin,^41^ may alter TLR ligand recognition. Cytokines, e.g., IL-4 or IL-13, may also suppress TLR-mediated signaling pathways.^85^ Commensal intolerance, i.e., exaggerated immune responsiveness of TLRs toward commensals, may occur as a consequence of endogenously or exogenously induced disturbance of any TLR-dependent signaling mechanisms of commensal tolerance. Positive regulators may enhance proinflammatory TLR signaling via NF-κB, such as the scaffold protein AKAP13.^86^ Inflammation in IBD may result from persistent commensal intolerance because of altered pattern recognition and TLR signaling. However, a more comprehensive analysis of the diverse TLR-dependent signaling mechanisms of commensal-mediated suppression of intestinal inflammation and how imbalance in positive versus negative signaling regulators may contribute to the pathogenesis of human IBD is needed.

### Pathogens

Episodes of *Salmonella/Campylobacter* gastroenteritis have been associated with increased risk of developing IBD.^87^ “Loss-of-function” mutations in the TLR4 gene can predispose to these Gram-negative bacteria and increase susceptibility to enteric infection—which may represent an essential disease trigger in IBD pathogenesis. Pathogenic infections may change the commensal composition and disrupt commensal tolerance. *Campylobacter jejuni* may directly promote the internalization and translocation of commensal bacteria.^88^ Host deficiency in bacterial clearance may allow conventional or opportunistic pathogens to provoke and sustain inflammatory responses via TLRs (and other PRRs), exacerbating or complicating underlying IBD, which may explain the high prevalence of persistent or recurrent infections in patients with chronic IBD.^89^ Viral pathogens, such as cytomegalovirus (CMV; a known risk factor in refractory and complicated IBD^90^), may also manipulate TLR-mediated immunity by priming TH1/TH17-dependent immune responses to the commensal microbiota.^91^ Inflammatory damage may be augmented by exaggerated maladaptive TLR responses through infection with attaching/effacing pathogens, as shown for the murine pathogen *Citrobacter rodentium*.^92^ Certain pathogenic microbes appear to have the capacity not only to avoid, but directly interfere with signaling components of the innate immune system, thereby subverting host defense mechanisms for their own virulent purposes (reviewed in Ref.^93^). Identification of subversion mechanisms that result in or from aberrant innate immunity of the intestinal mucosa may help to understand the differential influences of distinct (facultative/obligate) pathogens in IBD pathophysiology.

### Exogenous Injury Signals

Any mucosal insult of the intestine may result in tissue damage and activation of the innate and adaptive immune systems, followed by cell recruitment, proliferation, and migration, ultimately leading to wound healing. Deficient TLR signaling may imbalance commensal-dependent homeostasis, facilitating injury and leading to inflammatory disease. Dextran sulfate sodium (DSS)-induced colitis represents a well-established “damage”-model of acute chemically induced toxic injury in crypt colonic epithelial cells initiating inflammatory responses in the lamina propria. Injury may allow luminal TLR ligands to access the lamina propria and recruited primary immune cells to respond strongly to these ligands, thus triggering tissue destruction. Mice deficient in TLR2/3/4/5/9 or MyD88 exhibit delayed or diminished tissue repair responses during acute DSS-induced intestinal damage.^94^–^99^ Accordingly, systemic administration of a TLR4-blocking antibody impairs restoration of tissue integrity during DSS-colitis, despite limiting exaggeration of acute inflammatory responses induced by recruited cells.^100^ Several recent studies suggest that TLR signaling exerts many important cytoprotective functions in the intestinal epithelium (and adjacent cell subsets) which are required for barrier preservation, cell survival and stability, and restitution, including, e.g., inhibition of apoptosis, migration, and proliferation.

TLR2 is the only TLR identified so far to be capable of directly modulating the complex network of closely arranged tight (TJ) and gap junctions (GJ) of the intestinal epithelium. We have previously shown that stimulation of TLR2 rapidly enhances transepithelial resistance of the IEC barrier via PKC-α/δ by apical redistribution of ZO-1, a major TJ component.^101^ ZO-1 binds to the key gap junctional protein connexin-43 (Cx43), thus enhancing assembly and stabilization of gap junctional intercellular communication (GJIC)—an essential mechanism for cellular and tissue homeostasis. GJIC coordinates cell–cell passage of ions and small metabolites, regulating cell proliferation, migration, and differentiation (<1 kDa). In subsequent studies we demonstrated that inflammatory stress induces early and complete TJ/GJ-loss of the IEC barrier in the absence of TLR2, which is not evident in the presence of TLR2.^97^,^102^ Treatment with the TLR2 ligand PCSK protects TJ/GJ-associated integrity and decreases intestinal permeability, leading to significant amelioration of all clinical signs in acute DSS-induced colonic inflammation during the recovery phase.^97^ IEC-specific deficiency of Cx43 by mucosal RNA interference leads to abrogation of TLR2-mediated IEC restitution and delays wound closure in acute inflammatory stress-induced injury in the intestine.^102^ Collectively, these data show that TLR2 protects the local mucosa by preservation of TJ/GJ-associated architecture and GJIC between juxtaposed IEC during acute intestinal damage.

The PI3K/Akt-pathway controls cell survival, which plays a critical role in maintaining mucosal homeostasis. Stimulation with TLR2/5 ligands induces significant phosphorylation of Akt and its downstream substrates via MyD88 in IEC, thus essentially limiting proinflammatory stress signaling through the p42/p44 Mapk/p38 pathways.^97^,^103^,^104^ As a result, absence of TLR2/5 leads to deficient antiapoptotic IEC protection against toxic DSS-mediated damage,^97^,^99^ which further compromises TJ-associated barrier integrity and perpetuates intestinal inflammation. A different antiapoptotic pathway has recently been defined for epithelial TLR4 and TLR9 activation, upregulating expression of Cox-2,^105^,^106^ which is known to suppress apoptosis through PGE2 production in the gastrointestinal tract. TLR4-mediated production of hyaluronic acid may also induce macrophage Cox-2 and thus exert antiinflammatory effects during DSS colitis.^107^ Mesenchyme-derived PGE2 preserves proliferation of crypt progenitor cells via MyD88, which is essential for IEC restitution during acute DSS injury.^108^ Of note, it was recently proposed that DSS may act as a direct alarmin for TLR4,^107^ but whether DSS truly binds to TLR4 remains to be determined.

We have previously identified another, so far unappreciated, commensal-mediated antiapoptotic mechanism in the intestinal mucosa.^17^ Goblet cell (GC)-derived trefoil factor 3 (TFF3) plays a major role in wound healing and barrier repair of the intestinal mucosa.^109^ We showed that TLR2 acts to control terminal GC differentiation by selectively regulating TFF3 expression in the colon, thus conferring antiapoptotic protection of the intestinal mucosa against acute inflammatory stress-induced damage. TLR2 deficiency results in an innate immune defect of GC-derived TFF3, contributing to exacerbation of mucosal apoptosis and associated leukocyte influx during acute DSS injury, which can be reversed by supplementation with recombinant TFF3 peptide.^17^ Taken together, several TLR-dependent mechanisms that enhance IEC wound healing (barrier integrity, survival, cell–cell communication, proliferation, and migration) during acute exogenously induced mucosal injury have functionally linked products of commensal bacteria to innate immune protection of the intestine.

### Endogenous Danger Signals

Endogenous mediators and ligands may modulate TLR responsiveness and induce aberrant activation of TLRs under pathophysiological conditions. During tissue injury and inflammation, impaired “self” versus “nonself” discrimination by aberrant TLRs may lead to self-directed immune responses in autodysregulatory loops. For instance, the “alarmin” HMGB1, which is actively secreted by immune cells and passively released by necrotic nonimmune cells during colonic injury,^110^ can aggravate proinflammatory responses through activation of multiple TLRs.^111^ Excessive TLR activity may induce proapoptotic signaling pathways in primary immune cells. Apoptotic signals, in combination with TLR engagement, may perpetuate an innate IL-17 response,^112^ thus contributing to further tissue damage in IBD. However, development of TH17 polarization does not entirely depend on TLR signaling, as it was recently shown that ATP, a potent stimulus for NOD-like receptor (NLR)P3 inflammasome, triggers commensal-mediated TH17 responses in MyD88/TRIF-deficient mice.^113^ These findings highlight the complexity and diversity of innate immunity in the intestinal mucosa, which involves not only TLRs.

Danger signals can induce imbalances of intracellular homeostasis. Such disturbances may cause accumulation of unfolded proteins in the ER lumen, leading to an evolutionarily conserved cell stress response which, if not resolved, triggers cell death. Severe or prolonged ER stress has been implicated in initiation and/or perpetuation of intestinal inflammation and may represent a primary or secondary factor in the pathogenesis of IBD.^114^–^116^ The close proximity to the ER makes the TLR apparatus especially vulnerable to subcellular imbalances caused by pathophysiological levels of ER stress and aberrant activation of the unfolded protein response during chronic intestinal inflammation. Intestinal epithelial ER dysfunction as a primary defect or secondary to the inflammatory milieu may lead to misfolding and retention of newly synthesized TLRs and associated coreceptors that critically rely on this protein quality control machinery, as previously shown for MD-2 glycoprotein.^41^ Aberrant TLR/MD-2 accumulation in the ER could provide a means for limiting host reactivity by dampening ligand responsiveness. Conversely, IEC disequilibrium based on ER stress may turn other TLRs overly reactive to the resident microbiota (such as TLR5),^116^ which may exacerbate colitis. However, TLRs are also capable of initiating distinct cell survival mechanisms to protect against exaggerated ER stress. For instance, the continuous presence of the TLR4 ligand LPS may result in a state of ER hyporesponsiveness by suppressing the proapoptotic C/EBP homologous transcription factor (CHOP),^117^ thus decreasing cellular sensitivity to cytotoxic stress, e.g., in DSS-induced acute colitis, as outlined above.^118^ On the other hand, deficiency in TLR4 may reciprocally promote conditions of direct or indirect ER stress^119^ which may contribute to aggravation of colitis. Emerging evidence points to autophagy as a fundamental component of the innate immune process of cell survival against ER stress. The importance of deregulated autophagy in IBD pathogenesis has recently been elucidated by the association of genetic variants in ATG16L1 and IRGM with increased susceptibility to CD.^120^–^122^ Upon detection of nonself ligands, distinct TLRs may induce the autophagy machinery.^123^ Conversely, autophagy may transfer TLR agonists to the lysosomal pathway, thereby allowing recognition by endosomal TLRs.^124^ However, direct functional evidence is lacking that TLR ligands may modulate autophagy in the intestinal mucosa. Future studies are needed to dissect the precise molecular mechanisms of cause and effect that differentially influence the complex interdependence between ER stress and TLRs in the healthy versus inflamed intestinal mucosa in IBD. It must be clarified whether defects in the TLR pathways contribute to autophagic deficiency in IBD, or vice versa, thus promoting intestinal disease through impaired bacterial clearance and prolonged immune activation due to ER stress.

### Food Antigens

Shifts in the commensal composition may increase the ability of the microbiota to break down indigestible fiber into short-chain fatty acids,^125^ which may modulate innate immunity by downregulating TLR4 in IEC.^126^ High oral iron supplementation aggravates acute DSS colitis,^127^ presumably through aberrant induction of the TLR4-TRIF pathway in LPMNC, resulting in enhanced LPS-mediated proinflammatory cytokine expression.^128^ More studies will need to systematically determine the implications of different dietary antigens on mucosal TLR regulation and function with the goal of gaining insight into the mechanisms how these pleiotropic (co-)factors may possibly affect initiation, persistence, and relapses in human IBD.

## GENETICS

Genetic variations in TLRs may alter host–commensal interactions. A defect in TLRs may influence ligand recognition, mucosal immune tolerance, and commensal composition, leading to innate/adaptive immune hypo- or hyperreactivity. Several studies have evaluated the functional impact of TLR polymorphisms in IBD susceptibility and/or progression. None of these relatively rare TLR variants were captured as “major hits” by the recent genome-wide association scans. Individual TLR variants may have greater impact in terms of explaining differences in phenotype severity rather than providing predictions of disease risk in IBD.

### TLR1/2/4/5/6/9

A number of risk variants in the TLR1/2/6 genes have been associated with distinct disease phenotypes of IBD. UC patients with the polymorphisms TLR1-R80T and TLR2-R753Q appear to exhibit increased risk to develop pancolitis.^129^ The TLR2-R753Q mediates IEC dysfunction, failing to induce TFF3 synthesis in goblet cells^17^ and inhibiting GJIC through loss of Cx43 in enterocytes,^102^ which impairs restitution during wound healing. Thus, the TLR2-R753Q leads to more extensive disease due to impaired innate immune host defense in a subgroup of IBD. The cellular mechanistics of TLR1-R80T remains to be functionally defined. The single nucleotide polymorphism within TLR6, S249P, was associated with a slightly decreased incidence of proctitis in IBD.^129^

The TLR4 gene is localized on chromosome

9 (q32-33),^130^ a genomic region in which a CD susceptibility gene has been implicated.^131^ In active IBD, variant alleles in the TLR4 gene could induce functional dysregulation of the LPS receptor. “Gain-of-function” mutations could functionally exhibit proinflammatory effects in response to physiological concentrations of LPS. Two common mutations in the human TLR4 gene, D299G and T399I, have been observed to occur at a general frequency of between 6% and 10% in Caucasian populations.^132^ D299G polymorphism has been associated with CD as well as UC in several populations^133^–^135^; however, population studies reveal differences in geographical distribution.^136^ Increased susceptibility to IBD has been associated with the coexistence of TLR4 and/or caspase recruitment domain 15 (CARD15) and BPI^137^ mutated alleles.^138^ The presence of the D299G polymorphism in the absence of CARD15 mutations seems to be a particularly strong predictor of a stricturing disease phenotype in CD patients.^139^ Although D299G mutation (but not the T399I mutation) has been shown to interrupt TLR4-mediated LPS signaling in vitro,^140^ which could result from conformational changes,^141^ the functional phenotypic consequences remain unresolved in distinct cell types within the intestinal mucosa and in IBD. Highly variable TLR4 gene mutations have been identified in various mice strains that exhibit a broad distribution of different phenotypic responses to LPS, ranging from hyper- to hyposensitivity,^142^ suggesting that additional gene–gene interactions are involved.

It is possible that the D299G mutation may induce signaling disequilibrium of TLR5 leading to intestinal inflammation through “over”-recognition of flagellin. Hyperreactivity to flagellins was observed in sera from patients with CD.^67^,^143^,^144^ A recently identified dominant-negative TLR5 polymorphism (TLR5-stop), which leads to 75% loss of TLR5 function, reduces adaptive immune responses to flagellin, and, in Jewish cohorts, protects against the development of CD.^145^ Conversely, complete loss of TLR5 in mice may result in the development of spontaneous colitis via aberrant TLR4 signaling in response to changes in the commensal composition.^44^

The single-nucleotide polymorphisms (SNPs) -1237T/C and 2848A/G in the TLR9 gene were recently linked to CD-associated variants in CARD15, IL23R, and DLG5, thus differentially modulating CD susceptibility.^146^ It remains to be shown whether these SNPs may functionally impair TLR9 sensing of commensal DNA, leading to aggravation of inflammatory disease through Treg/Teff imbalance in the intestinal mucosa.^147^

In addition, a number of IBD-associated genetic defects, secondarily influencing the function and (patho)physiology of intestinal TLRs, have been identified and are associated with decreased or increased susceptibility to colitis:

### IL-10

IL-10 critically affects interactions between the microbiota and host defense of the gut in order to maintain mucosal homeostasis and prevent intestinal inflammation. A balanced microbiota plays an important role in the mucosal production of antiinflammatory IL-10. Commensal *Bacteroides* protects from colitis through the induction of interleukin-10-producing CD4+ T cells.^148^ Defects in the IL-10 gene disturb the bilateral host–symbiont relationship through alterations of mucosal immune responses, ultimately leading to intestinal inflammation. Mice that are deficient in IL-10 develop spontaneous TH1/TH17-driven enterocolitis,^149^,^150^ which resembles human IBD in many histopathological and immunological features and which is dependent on the presence of commensals.^151^ Loss-of-function mutations in the genes encoding the two polypeptide chains of the IL-10 receptor (IL-10RA or IL-10RB) have recently been associated with early onset hyperinflammation of the intestine in patients.^152^ Furthermore, a polymorphism in the IL-10 gene has been associated with increased risk to develop UC.^153^ Collectively, these data imply that absence of IL-10-mediated antiinflammatory immune responses may play an important role in the pathophysiology of human IBD.

The mechanisms by which IL-10 manages intestinal homeostasis and suppresses inflammation are diverse and include the following: maintaining the mucosal immune system in a partially unresponsive state and preventing the development of pathogenic T-cell-mediated responses to bacteria-specific antigens,^154^,^155^ sustaining the suppressive function of (Foxp3+) Tregs^156^ and restricting excessive immune responses by limiting uncontrolled generation of TH1/TH17 cells and production of proinflammatory cytokines (IFN-γ, IL-6, IL-12, IL-17, IL-23) in the lamina propria.^150^,^157^ Furthermore, IL-10 may negatively regulate cell signaling via TLR-MyD88 in an autocrine loop. The underlying mechanisms are not yet fully understood, but IL-10 may directly dampen MyD88-dependent proinflammatory signaling by ubiquitination and degradation of IRAK4 and TRAF6.^158^ Conversely, absence of MyD88 protects IL-10-deficient mice against the development of spontaneous enterocolitis by inhibiting downstream NF-κB-dependent TH1/TH17 polarization.^159^,^160^ Similarly, commensal-dependent expansion of colitogenic CD4+ TH17 cells is also impaired in Rag-2−/− mice transferred with MyD88−/− CD4^+^CD45RB^hi^ cells, resulting in significant delay in onset of spontaneous colitis.^27^,^28^

But our knowledge of how commensal-dependent TLRs, upstream of MyD88, may differentially regulate the phenotype of colitis in the IL-10-deficient host remains so far limited. Dual-association of germ-free IL-10−/− mice with the nonpathogenic commensal bacteria strains *E. coli/E. faecalis* induces severe colitis^155^ which is abolished in the absence of MyD88.^160^ Several TLRs (TLR2/3/4/5/9^161^,^162^) are selectively modulated by *E. coli/E. faecalis*. Two recent studies^163^,^164^ showed independently that TLR4 may not be the culprit in the pathogenesis of commensal-dependent colitis in IL-10−/− mice. On the contrary, the absence of TLR4 leads rather to an increase in intestinal inflammation in IL-10−/− mice with uncontrolled generation of aberrant IFN-γ- and IL-17-producing (Foxp3+) Tregs and altered control of IEC turnover.^163^ T-cell TLR4 can negatively regulate activation signals delivered by the T-cell receptor (TCR) through TRIF-dependent MKP3 signaling.^164^ These findings^163^,^164^ imply that essential TLR4 signals from the commensal microbiota help to limit propagation of colitic effector CD4+ T cells, and significantly ameliorate disease course in the context of IL-10 deficiency.

The contrasting effects of TLR4 and MyD88 in IL-10 deficiency are so far not fully understood. But it must be considered that TLR4 also signals through the MyD88-independent pathway via TRIF to induce type I IFN.^165^ TLR3/9-mediated production of type I IFN has been shown to protect against colitis in the IL-10-deficient mice by negatively regulating TH17 differentiation in the lamina propria.^166^,^167^ It remains to be investigated whether the antiinflammatory function of TLR4 signaling in the IL-10−/− host is linked to type I IFN induction via TRIF. It also remains to be resolved whether loss of mucosa-protective TLR4 leads to altered function of other TLRs (or PRRs) in the intestinal mucosa, which may be responsible for exacerbation of colitis in IL-10 deficiency. A role of non-TLR innate recognition cannot be ruled out. Although MyD88 serves as a scaffold adaptor protein at the crossroad of many common TLR-dependent signaling pathways, MyD88 is not TLR-specific. MyD88 converges with TLR-independent signaling modules, including focal adhesion kinase,^168^ IL-1R, and IL-18R.^169^ Future studies will need to dissect the mechanistic involvement of other TLRs (alone and in combination) as well as related innate immune sensors (NLRs) in commensal-dependent enterocolitis in the absence of IL-10. Using TLR ligands or drugs that selectively induce TRIF or type I IFN in the intestinal mucosa could have compelling potential to prevent or treat acute inflammatory flares in IBD patients with lack of IL-10 signaling.

### STAT3

However, the outcome of TLR4-mediated innate immune responses seems to be ambiguous, primarily resulting from variable involvement of affected signaling pathways and cell populations within the intestinal mucosa, depending on different IBD genotypes as well as diverse influences by the intestinal ecosystem. STAT3 plays a pivotal role in the control of innate immunity in the intestine. The gene region of STAT3 was recently linked to human IBD.^170^ STAT3 is a pleiotropic transcription factor activated by a variety of mucosa-/cytoprotective cytokines and growth factors. For instance, IL-22 mediates mucosal restitution of mucus-producing goblet cells via STAT3.^171^ Consequently, IEC-specific deletion of STAT3 leads to increased susceptibility to acute DSS colitis.^172^ Myeloid cell-specific deletion of STAT3 results in development of spontaneous enterocolitis in mice.^173^ Strikingly, crossing myeloid cell-specific STAT3-deficient mice with TLR4-knockout attenuates intestinal inflammation,^174^ implying that disruption of STAT3 expression leads to overly activated innate immune responses via TLR4 toward the resident microflora and interferes with the adaptive immune system by inhibiting induction of antigen-specific T-cell tolerance in the intestinal mucosa. In return, defective T-cell apoptosis may subvert signaling of, e.g., TLR5/9 (or other PRRs), further mobilizing the effector function of TH1 cells, leading to enhanced IFNγ and IL-12/IL-23 production and excessive cell proliferation, which aggravates and sustains injurious immunologic reactions in the inflamed intestine. Taken together, in the scenario of a genetic STAT3 defect, targeted inhibition of TLR4 recognition and signaling may represent a more desirable treatment goal in order to comprehensively abolish climaxed TH1-dominant inflammatory responses that are caused by long-standing, cell-specific innate and adaptive immune alterations to luminal antigens in the lamina propria.

### CARD15

CARD15 exerts antibacterial activity through defensins,^175^,^176^ thus critically influencing the commensal composition.^45^ Genetic variations of CARD15 have been linked to increased susceptibility to some types of CD^177^,^178^ and associated with defensin deficiency.^69^,^179^,^180^ Of note, CARD15 synergizes with TLRs to trigger TH2-type polarization,^181^ contributing to maintenance of TLR-mediated intestinal homeostasis by downmodulating exaggerated TH1-responses in the intestinal mucosa. Chronic CARD15 stimulation abrogates TLR-induced proinflammatory immune responses in a cell-type-specific manner through IRAK-M.^182^ Once TLR-tolerant, macrophages still remain responsive to stimulation via CARD15.^183^ Colonic lamina propria cells isolated from mice treated with a CARD15 ligand and cultured ex vivo exhibit reduced production of proinflammatory cytokines upon TLR stimulation, while the presence of a CD-associated CARD15 mutation fails to block TLR-mediated NF-κB-hypersensitivity in adenomatous polyposis coli (APC).^184^,^185^ Together, these observations highlight the importance of regulatory networks and synergy between TLR and CARD15 pathways in sustaining controlled innate immune responses to maintain normal mucosal homeostasis. Imbalanced interactions between CARD15 and TLR pathways could lead to disease by uncontrolled and excessive actions of the innate immune system. Genetic defects in CARD15 may lead to changes in the commensal composition due to defensin deficiency, which may disturb homeostatic TLR signaling, thus promoting inflammatory disease.

### MDR1α

Several variations of the ABCB1/MDR1 gene have been associated with increased susceptibility to UC.^186^ Mice deficient in MDR1α develop spontaneous chronic colitis that resembles human UC.^187^ Commensal bacteria drive the inflammatory process and changes in the commensal composition due to lack of the MDR1α transporter have recently been described,^188^ but the innate immune mechanisms functionally involved in modulating disease have not been well defined yet. CRX-526, a lipid A-mimetic that acts as a TLR4 inhibitor, blocks LPS-induced TNF-α, IL-12p70. and IL-6 in DCs and monocytes in vitro. Systemic administration of CRX-526 has been shown to effectively inhibit the development of chronic colitis in MDR1α-deficient mice, implying a potential role of aberrant TLR4 activation in perpetuating inflammatory responses in the presence of MDR1α defects.^189^ However, recent studies suggest that some lipid A mimetics may also display agonistic activity in vivo, despite antagonistic effects in vitro.^190^ The cellular mechanisms remain to be resolved in detail how CRX-526 could have exerted its beneficial effects in vivo. Furthermore, conventional housing conditions (e.g., *Helicobacter bilis*) may influence the clinical course of colitis in MDR1α-deficient mice.^191^,^192^ It remains to be directly shown whether selected microbial species due to changes of the commensal composition may subvert host immune responses in the context of MDR1α deficiency, thus possibly allowing pathogenic hyperactivation of TLR4 (and other TLRs).

The pathogenesis of pancolitis in MDR1α−/− mice involves a primary IEC barrier defect associated with alterations in IFNγ-responsive genes in the lamina propria.^187^,^193^ We have previously shown that prophylaxis with a TLR2 agonist delays spontaneous onset of chronic colitis in MDR1α-deficient mice raised under specific-pathogen-free conditions.^102^ Despite the presence of CC-chemokines and proinflammatory IFNγ in the mucosal milieu, treatment with the TLR2 ligand maintained IEC barrier integrity, by preventing TJ/GJ disruption and associated exaggeration of inflammatory responses in this genetically susceptible host.^102^ These findings demonstrate that TLR2-mediated preservation of IEC barrier function not only protects against acute inflammatory stress-induced mucosal damages but also prevents spontaneous chronic colitis. The continuous presence of the TLR2 agonist by long-term administration in relatively high concentrations may have elicited cross-tolerance toward the facultative-pathogenic commensal communities in MDR1α deficiency.^13^ Future studies will need to examine whether selective TLR2 activation may also exert mucosa-protective effects in other murine models of colitis (such as IL-10−/−; STAT3-mutant). The contribution of additional antiinflammatory responses mediated by TLR2 on lamina propria mononuclear cells must be elucidated in detail.^194^

## COLITIS-ASSOCIATED CANCER IN IBD

Commensals may trigger colon tumor formation through amplification of TH17 responses during colitis.^195^ Recent studies have highlighted an important role of aberrant TLR signaling in promoting colitis-associated cancer. TLRs are expressed on colon cancer cells and neighboring cells in the microenvironment. Studies using chemically induced colon carcinogenesis in chronic DSS colitis demonstrate that hyperproliferative TLR signals may influence the course of inflammation-associated colorectal cancer. TLR4 signaling on IEC is necessary for recruitment and activation of Cox-2-expressing macrophages that promote colorectal tumor growth through PGE2.^196^ TLR4-deficient mice which fail to produce PGE2 are protected from colon cancer in this model,^197^ whereas mice deficient in SIGIRR, a negative regulator for TLR4 signaling, demonstrate increased susceptibility.^77^,^78^ However, while TLR4 activation promotes proliferative tumor-responses in IEC, it may induce antitumor responses in cytotoxic T cells.^198^ This finding implies an ambiguous role of TLR4 in tumor development, depending on the predominant cell types involved.

It remains unclear how innate immune dysfunction contributes to tumor development through impairment of cancer cell stem cell differentiation and survival via the Wnt-APC-β-catenin cascade. A possible indirect link between MyD88 and the tumor suppressor gene APC has recently been reported.^199^ MyD88 null mice that also carry a germline mutation in the APC gene exhibited regression of spontaneous development of pretumoric adenomas, however, predominantly in the small intestine, which argues against a primary role of TLR signaling in the Apc^Min/+^ tumor model, as the bacterial load is normally very low in this part of the gastrointestinal tract. Of note, MyD88−/−/Apc^Min/+^ mice exhibited increased IEC apoptosis and it is possible that deficiency of MyD88 impaired stability of its death domain-partner FADD to enhance recruitment of caspase 8, promoting IEC turnover and delaying tumor progression.

It will be important to examine how aberrant modulation of TLRs contributes to initiation and progression of colitis-associated neoplasia in the context of IBD-associated gene deficiency, such as IL-10. A brief report previously proposed that absence of TLR4 drives aggressive development of spontaneous colitis-associated adenocarcinoma in IL-10−/− mice,^200^ but detailed investigations of the underlying mechanisms are so far lacking. Dissecting the direct and indirect interactions between components of the “environment-genetics-immunity”-triad and TLRs that trigger cancer pathogenesis in human IBD may help to develop novel immunomodulators that mediate effective cytotoxicity in cancer cells only and induce distinct antitumor responses in the microenvironment of the intestinal mucosa.

## CONCLUSIONS

An important role for TLR signaling (summary in Table 1) in the pathogenesis of IBD has been established through many studies over the last decade. The impact of TLR signaling on commensal–host interactions appears to be context-dependent. Environment, genetics, and host immunity modulate TLRs in the intestinal mucosa (Fig. 1). Conversely, mucosal TLR signaling influences outcome of environmental signals, genetic functions, and immune responses in the intestine. There is an important dichotomy in TLR regulation and function between healthy and inflamed intestinal mucosa, reflecting a fine line between host protection and destruction. In the healthy host, basal TLR signaling is significantly involved in protective host defense and tissue repair responses, crucially maintaining mucosal and commensal homeostasis. In the IBD-susceptible host, aberrant TLR signaling may contribute to destructive host responses and chronic inflammation, disturbing mucosal and commensal homeostasis and leading to many different clinical phenotypes (summary in Table 2). Hyperactivation of the adaptive immune system, secondary to TLR deficiency, may drive tissue damage and progressive inflammation in IBD. Characterization of different IBD-associated gene defects have highlighted fundamental, defining variability in TLR regulation and function, dependent on disease processes and predominant cell type involvement in the intestinal mucosa (summary in Table 3). Further advances in our mechanistic understanding of this multilayered interplay (Fig. 1) between components of the “environment-genetics-immunity” triad and TLRs (and other PRRs) will be central to future progress in 1) elucidating geno-/phenotype correlations in the pathogenesis of IBD and colitis-associated colorectal cancer, and 2) identifying novel approaches that may exploit innate immune functions as a means to prevent and/or treat IBD and related systemic manifestations.

**TABLE 1:** Toll-like Receptors (TLRs)

**TLR structure and signaling**
•13 mammalian, type I transmembrane glycoreceptors (10 in humans; 12 in mice) with divergent LRR-ectodomain and conserved intracellular TIR domain
•recognition of alarm patterns or signals:
a)microbiota/viral-associated (commensal/pathogen)
b)damage-associated (endogenous/exogenous)
•downstream activation of pro-/antiinflammatory cytokines and chemokines and link to adaptive immunity through at least 5 different adaptor proteins
**Regulatory dichotomy in TLR expression and function between health and IBD**
•constitutively or inducibly expressed throughout the whole GI tract by a wide variety of cell types, including IEC lineages, myofibroblasts, monocytes/macrophages, DCs and T cells
•healthy intestine:
a)TLRs are present only in small amounts
b)negative regulators maintain basal state of activation and prevent prolonged and excessive TLR signaling
•diseased intestine:
a)distinct TLRs are significantly upregulated in certain cell subsets in intestinal mucosa
b)positive regulators initiate aberrant state of activation and allow uncontrolled TLR signaling

See text for details and references.

**TABLE 2:** (Patho)physiology of TLRs in the Intestinal Mucosa

**Physiological effects of normal TLR signaling in the healthy GI tract**
•Integrity of commensal composition and commensal tolerance
•Protection of intestinal epithelial/mucosal barrier function, accelerated wound healing
•Control of Treg↔Teff – balance in the intestinal mucosa
*→ Maintenance of commensal and mucosal homeostasis*
**Pathophysiological effects of aberrant TLR signaling in IBD**
•Changes in commensal composition and commensal intolerance
•Impairment of intestinal epithelial/mucosal barrier function, delayed wound healing
•Promotion of Treg↔Teff – imbalance in the intestinal mucosa
*→ Disturbance of commensal and mucosal homeostasis*

See text for details and references.

**TABLE 3:** Genetic Influences on TLRs in the Intestinal Mucosa

**Primary genetic defects in TLR function, influencing IBD susceptibility/progression**
•TLR1-R80T: associated with UC pancolitis; cellular dysfunction unclear
•TLR2-R753Q: associated with UC pancolitis; impairs IEC restitution and communication (TFF3↓; GJIC↓)
•TLR4-D299G: associated with increased susceptibility to IBD; interrupts LPS signaling
•TLR5-stop: associated with decreased susceptibility to IBD; reduces adaptive immune responses to flagellin
•TLR6-S249P: associated with decreased susceptibility to IBD proctitis; cellular dysfunction unclear
•TLR9-(-1237T/C), -(2848A/G): associated with CD-variants CARD15, IL23R, DLG5; cellular dysfunction unclear
**Murine defects in IBD-associated genes, secondarily influencing TLR function**
•IL10-/-: TLR4 limits propagation of colitic effector CD4+ T cells, thus ameliorating disease course
•STAT3-mutant: TLR4 triggers induction of spontaneous enterocolitis
•CARD15-/-: failure to block TLR-hyperactivity; changes in commensal composition due to cryptidin deficiency
•MDR1α-/-: lipid A-mimetic blocks induction of colitis; TLR2 ligand delays onset of colitis

See text for details and references.
